# Efficacy of mitral valve repair in combination with coronary revascularization for moderate ischaemic mitral regurgitation: a systematic review and meta-analysis of randomized controlled trials

**DOI:** 10.1097/JS9.0000000000001277

**Published:** 2024-03-19

**Authors:** Xin Li, Biao Hou, Shuwen Hou, Wenjian Jiang, Yuyong Liu, Hongjia Zhang

**Affiliations:** a Department of Cardiac Surgery Center, Beijing Anzhen Hospital, Beijing Institute of Heart, Lung, and Blood Vascular Diseases, Capital Medical University, Chaoyang district, Beijing; b Department of Cardiovascular Surgery, The First Affiliated Hospital of Anhui Medical University, Shushan district, Hefei, China

**Keywords:** coronary revascularization, mitral valve repair, moderate ischaemic mitral regurgitation, randomized controlled trials, systematic review and meta-analysis

## Abstract

**Background:**

The efficacy of mitral valve repair (MVR) in combination with coronary artery bypass grafting (CABG) for moderate ischaemic mitral regurgitation (IMR) remains unclear. To evaluate whether MVR + CABG is superior to CABG alone, the authors conducted a systematic review and meta-analysis of existing randomized controlled trials (RCTs).

**Methods:**

The authors searched PubMed, Web of Science, and the Cochrane Central Register of Controlled Trials for eligible RCTs from the date of their inception to October 2023. The primary outcomes were operative (in-hospital or within 30 days) and long-term (≥ 1 year) mortality. The secondary outcomes were postoperative stroke, worsening renal function (WRF), and reoperation for bleeding or tamponade. The authors performed random-effects meta-analyses and reported the results as risk ratios (RRs) with 95% CIs.

**Results:**

Six RCTs were eligible for inclusion. Compared with CABG alone, MVR + CABG did not increase the risk of operative mortality (RR, 1.244; 95% CI, 0.514–3.014); however, it was also not associated with a lower risk of long-term mortality (RR, 0.676; 95% CI, 0.417–1.097). Meanwhile, there was no difference between the two groups in terms of postoperative stroke (RR, 2.425; 95% CI, 0.743–7.915), WRF (RR, 1.257; 95% CI, 0.533–2.964), and reoperation for bleeding or tamponade (RR, 1.667; 95% CI, 0.527–5.270).

**Conclusions:**

The findings of this meta-analysis suggest that MVR + CABG fails to improve the clinical outcomes of patients with moderate IMR compared to CABG alone.

## Graphical abstract

**Figure:**
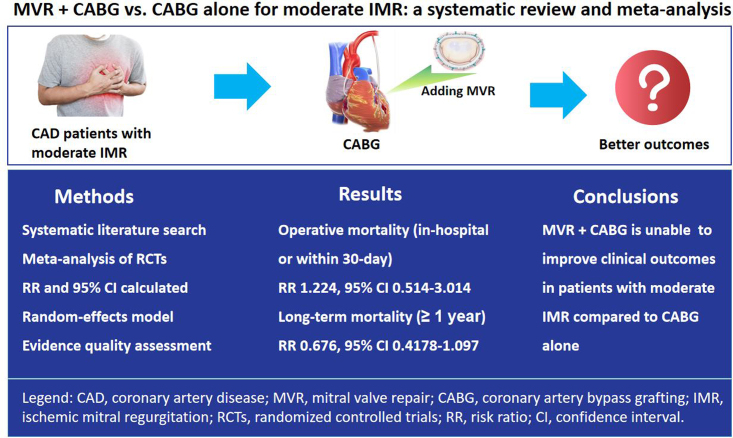

## Introduction

Highlights

- Whether mitral valve repair (MVR) + coronary artery bypass grafting (CABG) provides benefits for the treatment of moderate ischaemic mitral regurgitation (IMR) remains unclear. The ongoing debate primarily arises from the lack of strong and reliable evidence.
- The findings from this meta-analysis of randomized controlled trials suggest that MVR + CABG fails to improve the clinical outcomes of patients with moderate IMR compared with CABG alone.
- MVR + CABG may offer a potential long-term survival advantage compared with CABG alone for patients with IMR with severe left ventricular dysfunction (LVEF < 40%).

Patients with myocardial infarction (MI) frequently experience left ventricular (LV) dilation and adverse remodelling, resulting in mitral complex incompetence and ultimately leading to mitral regurgitation (MR)^1–3^. The incidence rate of moderate or severe ischaemic MR (IMR) in this patient population is reported greater than 10%^4^. Significant IMR indicates severe LV dysfunction and is associated with poor long-term survival^5,6^. Clinical guidelines recommend that in patients with severe IMR, combined mitral valve surgery is reasonable when coronary artery bypass grafting (CABG) is undertaken for treating myocardial ischaemia^7,8^. However, the benefit of adding mitral valve repair (MVR) to CABG in patients with moderate IMR remains unclear.

To address this clinical gap, the POINT study was the first randomized controlled trial (RCT) on this topic^9^. It revealed that although MVR + CABG is more effective in improving heart failure symptoms and cardiac functional status compared with CABG alone, it has no significant advantage in terms of survival. However, subsequent studies have yielded varying results, and a unified conclusion has not yet been reached. Therefore, the present study aimed to provide high-quality evidence for clinical practice by conducting an up-to-date systematic review and meta-analysis of RCTs to evaluate the outcomes of MVR + CABG versus CABG alone in patients with moderate IMR.

## Materials and methods

### Study design

The present systematic review has been registered in the International Prospective Register of Systematic Reviews. We reported this work in line with PRISMA (Preferred Reporting Items for Systematic Reviews and Meta-Analyses guidelines, Supplemental Digital Content 1, http://links.lww.com/JS9/C147 Supplemental Digital Content 2, http://links.lww.com/JS9/C148) and AMSTAR (Assessing the methodological quality of systematic reviews, Supplemental Digital Content 3, http://links.lww.com/JS9/C149) Guidelines^10,11^.

### Data sources and search strategy

Electronic databases, including PubMed, Web of Science, and the Cochrane Central Register of Controlled Trials, were systematically searched from their inception date to October 2023. The query words included the following: ischaemic, functional, secondary, mitral regurgitation, mitral incompetence, mitral insufficiency, mitral dysfunction, randomized, and clinical trials. There were no restrictions on the language or year of publication.

### Selection criteria

The authors independently assessed the titles and abstracts of the retrieved citations. If a study was considered potentially eligible, the full text was carefully reviewed. Any disagreements regarding the final included studies were resolved through discussion and consensus. The selection criteria were^1^: studies using an RCT design^2^, studies assessing patients with a diagnosis of moderate IMR, and^3^ studies reporting at least one primary outcome. Studies were excluded if^1^ included patients with mixed MR etiologies^2^, had no available data for analysis, or^3^ did not include combined CABG.

### Data collection and management

The authors independently extracted data according to the “Participants, Interventions, Comparisons, and Outcomes” principle. Any discrepancies in data extraction were resolved through discussion and consensus. The extracted information included the first author, region, publication year, trial name, sample size, age, sex composition, intervention, comparison, and clinical outcomes. The extracted data for synthesis were transferred to an Excel (Microsoft Corp.) sheet and double-checked for accuracy.

### Risk-of-bias assessment

The authors used the Cochrane risk-of-bias (ROB) tool version 2.0 (https://www.riskofbias.info) to independently assess the risk of bias for each included study. The ROB tool 2.0 comprises five domains: randomization process, deviations from intended interventions, missing outcome data, outcome measurement, and selection of the reported result. Within each domain, there were signalling questions that the authors needed to answer based on their judgment. The risk of bias in each domain was categorized as “low,” “high,” or “some concerns.” Any discrepancies in judgment were resolved through discussion and consensus.

### Outcomes

The primary outcomes were operative and long-term mortality. Operative mortality was defined as death occurring during the hospital stay or within 30 days of surgery. Long-term mortality was defined as death occurring during the study’s longest follow-up period (minimum 1 year). The secondary outcomes were postoperative stroke, worsening renal function (WRF; defined as acute kidney injury, renal failure, or need for haemodialysis), and reoperation for bleeding or tamponade.

### Statistical analyses

For dichotomous data, we calculated the risk ratios (RRs) and corresponding 95% CIs. Heterogeneity was quantitatively assessed using the I^2^ statistic. According to the guidelines of the Cochrane Handbook for Systematic Reviews of Interventions (https://training.cochrane.org/handbook), an I^2^ value of 0–40% might not be important, 30–60% may represent moderate heterogeneity, 50–90% may represent substantial heterogeneity, and 75–100% suggests considerable heterogeneity. A random-effects model was employed for data synthesis, regardless of the level of heterogeneity. However, if I^2^ was greater than 50%, we planned to explore the potential causes of heterogeneity using subgroup analyses or meta-regression. Publication bias for each outcome was assessed by conducting a funnel plot. The symmetry of the funnel plot was quantitatively evaluated using Begg’s and Egger’s tests. For the primary outcomes, sensitivity analyses were performed to assess the robustness of the pooled results. The following approaches were applied^1^: using the odds ratio as the summary statistic and^2^ excluding each included study individually. Additionally, the effects of treatment strategies on the primary outcomes in different subgroups were explored. The following subgroups were considered^1^: age older than or equal to 65 and younger than 65 years and^2^ LV ejection fraction (LVEF) greater than or equal to 40% and less than 40%.

Analyses were performed using the Comprehensive Meta-Analysis version 3.0 (Biostat) and Stata version 12.0 (StataCorp). A two-sided *P* value of less than 0.05 was considered statistically significant.

### Certainty of evidence

Evidence quality was graded as high, moderate, low, or very low using the Grading of Recommendations, Assessment, Development, and Evaluations (GRADE) scoring system^12^. The system commences by assessing the study design, followed by considering five factors (risk of bias, imprecision, inconsistency, indirectness, and publication bias) that may lead to a decrease in evidence quality and three factors (large effect, dose-response, and all plausible residual confounding) that may lead to an increase in evidence quality. The initial evidence quality of randomized trials is considered high and may be upgraded or downgraded based on the assessment of the above factors. GRADEpro GDT (https://www.gradepro.org) was used to complete evidence quality assessment.

## Results

### Results of the search

**Figure 1:**
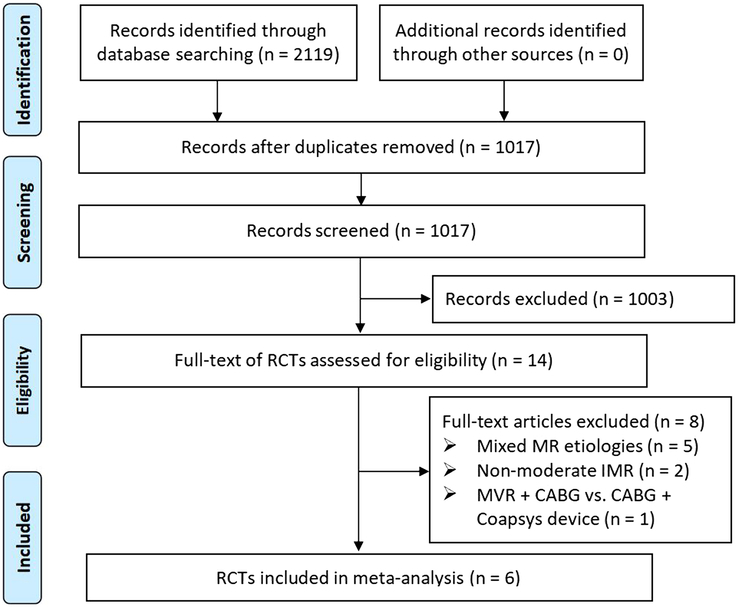
PRISMA flow diagram for this study. CABG, coronary artery bypass grafting; IMR, ischaemic mitral regurgitation; MR, mitral regurgitation; PRISMA, Preferred Reporting Items for Systematic Reviews and Meta-Analyses; RCTs, randomized controlled trials.

A database search was completed in October 2023 (Tables S1 and S2, Page 2, Supplemental Digital Content 4, http://links.lww.com/JS9/C150). In total, 2119 citations were found, out of which 2105 duplicates, meta-analyses, reviews, case reports, non-RCTs, and irrelevant studies were eliminated. After carefully reviewing the full text of the remaining studies, eight studies^13–20^ were excluded for the following reasons^1^: patients had mixed MR etiologies^2^, the grade of MR was severe, and^3^ the comparison was MVR + CABG versus CABG + LV reshaping. Ultimately, six RCTs^9,21–25^ were included in the quantitative meta-analysis. The search, screening, and study selection processes are illustrated in Fig. 1.

### Characteristics of the included studies

The six included RCTs were published between 2009 and 2020 and conducted in seven different countries, with two of them being multi-centre trials. The sample sizes of the included RCTs ranged from 31 to 301, and 626 patients (309 who underwent MVR + CABG and 317 who underwent CABG alone) were used for data synthesis. Among these patients, 71.4% were male and 75.6% had a history of MI. Table 1 presents an overview of the main characteristics and demographics of the included RCTs.

**Table 1:** Characteristics of the RCTs included in this meta-analysis.

Study (Acronym)	Fattouch 2009 (POINT)^9^		Chan 2012 (RIME)^22^		Bouchard 2014^21^	
Design	Single-centre RCT		Multicenter RCT		Single-centre RCT	
Region	Italy		UK and Poland		Canada	
Study period	2003–2007		2007–2011		2002–2008	
Primary endpoints	NYHA functional class, LVESD, LVEDD, LVEF		Peak oxygen consumption at 1 year		Left ventricular dimension changes at 1 year	
Group	MVR + CABG	CABG alone	MVR + CABG	CABG alone	MVR + CABG	CABG alone
No. participants	48	54	34	39	15	16
Male, *n* (%)	30 (52.5)	35 (64.8)	25 (74)	29 (74)	12 (75)	14 (88)
Age (years)	64 ± 6	66 ± 7	70.9 ± 10.5	70.4 ± 7.9	69 ± 7	65 ± 12
LVEF (%)	42 ± 10	43 ± 9	40 ± 17.3	40.3 ± 16.1	45.7 ± 11.4	41.5 ± 17.4
HTN, *n* (%)	26 (54)	23 (42.5)	17 (50)	23 (59)	11 (73)	9 (56)
DM, *n* (%)	28 (58.3)	32 (59)	12 (35)	15 (38)	4 (27)	8 (50)
Previous MI, *n* (%)	48 (100)	54 (100)	25 (74)	28 (72)	9 (60)	12 (75)
ACC time (min)	88 ± 19	38 ± 8	99 ± 15	49 ± 11	94 ± 28	63 ± 29
CPB time (min)	112 ± 32	65 ± 17	150 ± 28	87 ± 28	116 ± 37	89 ± 31
Study (Acronym)	Smith 2014 (CTSN)^25^		Kareva 2019^23^		Khallaf 2020^24^	
Design	Multicenter RCT		Single-centre RCT		Single-centre RCT	
Region	US and Canada		Russia		Egypt	
Study period	2009–2013		NA		2014	
Primary endpoints	Left ventricular end-systolic volume index at 1 year		Postoperative clinical results and long-term survival		Postoperative clinical results and echocardiographic outcomes	
Group	MVR + CABG	CABG alone	MVR + CABG	CABG alone	MVR + CABG	CABG alone
No. participants	150	151	38	38	20	20
Male, *n* (%)	106 (70.7)	99 (65.6)	30 (79)	34 (89)	11 (55)	12 (60)
Age (years)	64.3 ± 9.6	65.2 ± 11.3	57.6 ± 10.0	57.4 ± 7.7	54.3 ± 4.9	53.9 ± 4.7
LVEF, *n* (%)	39.3 ± 10.9	41.2 ± 11.6	30.0 ± 7.1	31.0 ± 4.8	49 ± 4	51 ± 5
HTN, *n* (%)	NA	NA	NA	NA	12 (60)	9 (45)
DM, *n* (%)	76 (50.%)	66 (43.7)	29 (76)	28 (74)	12 (60)	11 (55)
Previous MI, *n* (%)	103 (68.7)	97 (64.2)	36 (95)	36 (95)	13 (65)	12 (60)
ACC time (min)	117 ± 35	75 ± 37	NA	NA	74 ± 8	43 ± 9
CPB time (min)	163 ± 55	107 ± 50	NA	NA	91 ± 10	56 ± 9

Values are presented as either mean ± standard deviation or a count with its corresponding proportion.

ACC, aortic cross clamping; CABG, coronary artery bypass grafting; CPB, cardiopulmonary bypass; DM, diabetes mellitus; HTN, hypertension; LVEDD, left ventricular end-diastolic diameter; LVEF, left ventricular ejection fraction; LVESD, left ventricular end-systolic diameter; MI, myocardial infraction; MVR, mitral valve repair; NA, not available; NYHA, New York Heart Association; RCT, randomized controlled trials.

### Risk of bias in included studies

**Figure 2:**
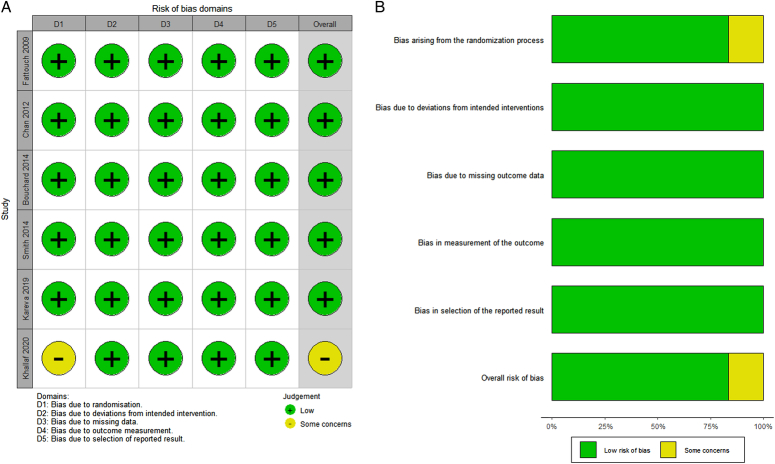
Risk-of-bias assessment for included RCTs. The graph displays (A) distribution of the bias for each study and (B) summary of the risk of bias. RCTs, randomized controlled trials.

Based on the evaluation of the ROB tool 2.0 (Figures S1 and S2, Page 4, Supplemental Digital Content 4, http://links.lww.com/JS9/C150), five RCTs were categorized as low risk. In contrast, one RCT^24^ was classified as having some concerns owing to the lack of a specific description of the randomization process by the study’s author. The ROB assessments of the included RCTs are shown in Fig. 2.

### Results of data synthesis

**Figure 3:**
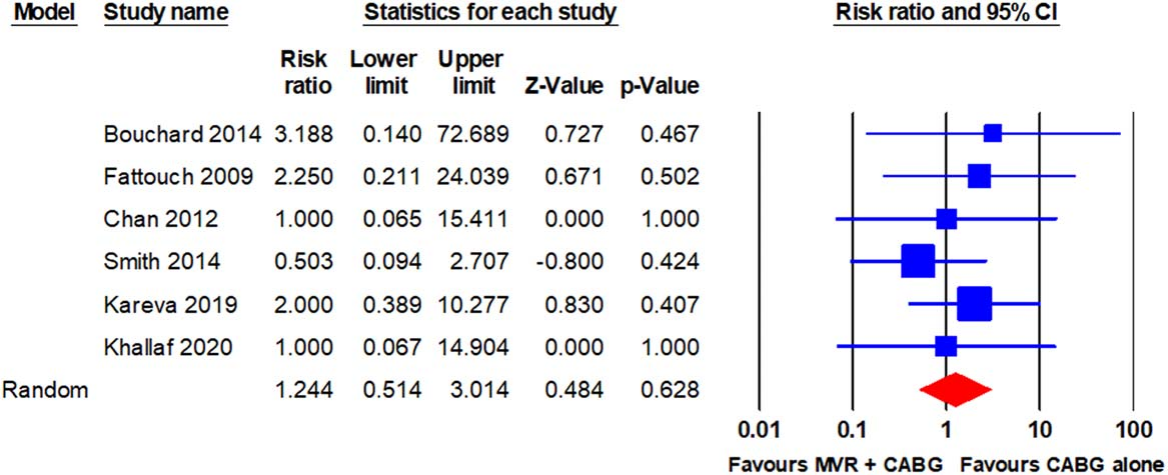
Forest plot for operative mortality. CABG, coronary artery bypass grafting; MVR, mitral valve repair.

Operative mortality was reported in all RCTs^9,21–25^. The pooled analysis using a random-effects model revealed no statistically significant difference in operative mortality between patients who underwent MVR + CABG and those who underwent CABG alone (11/309 vs. 8/317; RR, 1.244; 95% CI, 0.514–3.014; *P* = 0.628). The analysis revealed no significant heterogeneity (I^2^ = 0%). The forest plot is shown in Fig. 3.

**Figure 4:**
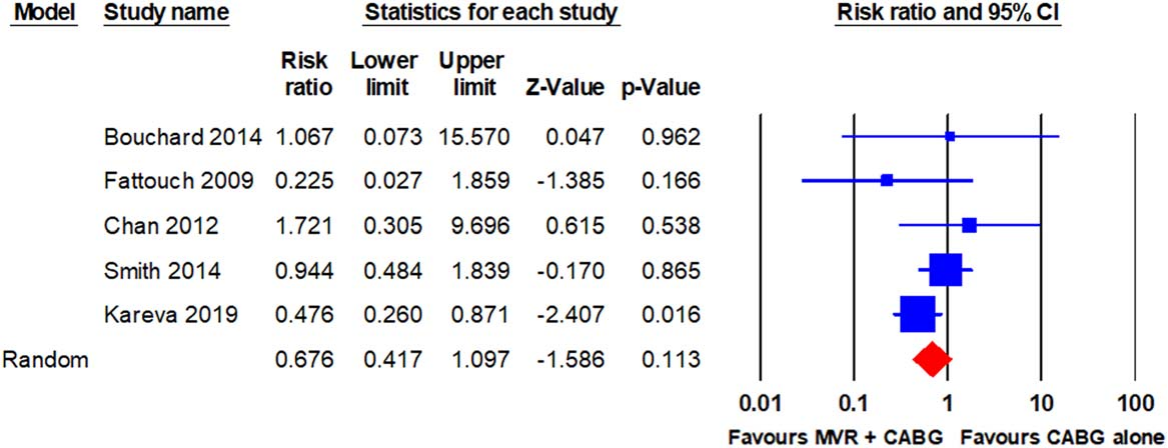
Forest plot for long-term mortality. CABG, coronary artery bypass grafting; MVR, mitral valve repair.

Long-term mortality was reported in five RCTs^9,21–23,26^. The pooled analysis using a random-effects model revealed no statistically significant difference in long-term mortality between patients who underwent MVR + CABG and those who underwent CABG alone (30/285 vs. 45/298; RR, 0.676; 95% CI, 0.417–1.097; *P* = 0.113). The analysis revealed minimal heterogeneity (I^2^ = 11.6%). The forest plot is shown in Fig. 4.

**Figure 5:**
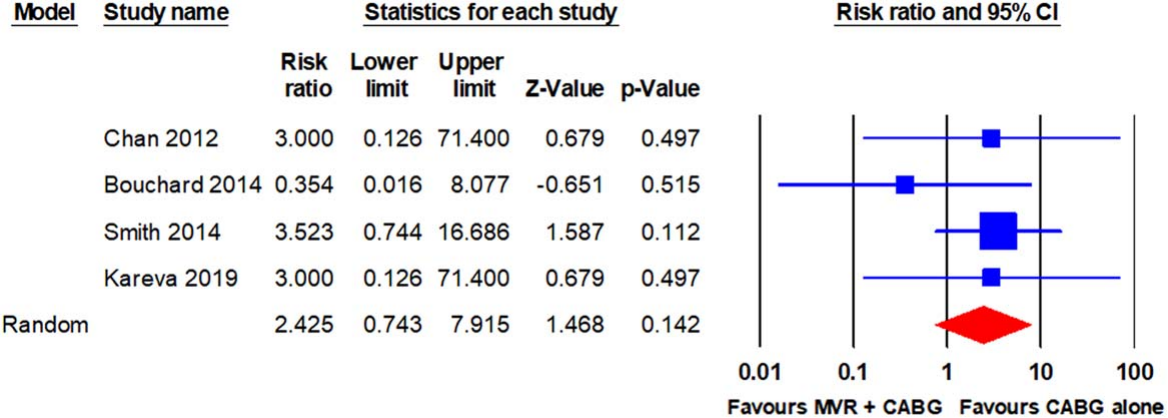
Forest plot for stroke. CABG, coronary artery bypass grafting; MVR, mitral valve repair.

Stroke was reported in four RCTs^21–23,25^. The pooled analysis using a random-effects model revealed no statistically significant difference in stroke between patients who underwent MVR + CABG and those who underwent CABG alone (9/241 vs. 3/243; RR, 2.425; 95% CI, 0.743–7.915; *P* = 0.142). The analysis revealed no significant heterogeneity (I^2^ = 0%). The forest plot is shown in Fig. 5.

**Figure 6:**
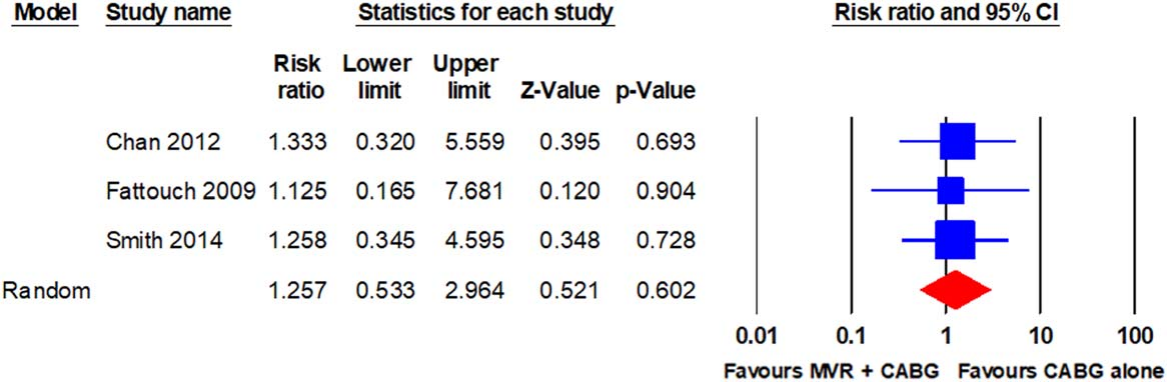
Forest plot for WRF. CABG, coronary artery bypass grafting; MVR, mitral valve repair; WRF, worsening renal function.

WRF was reported in three RCTs^9,22,25^. The pooled analysis using a random-effects model revealed no statistically significant difference in WRF between patients who underwent MVR + CABG and those who underwent CABG alone (11/236 vs. 9/243; RR, 1.257; 95% CI, 0.533–2.964; *P* = 0.602). The analysis revealed no significant heterogeneity (I^2^ = 0%). The forest plot is shown in Fig. 6.

**Figure 7:**
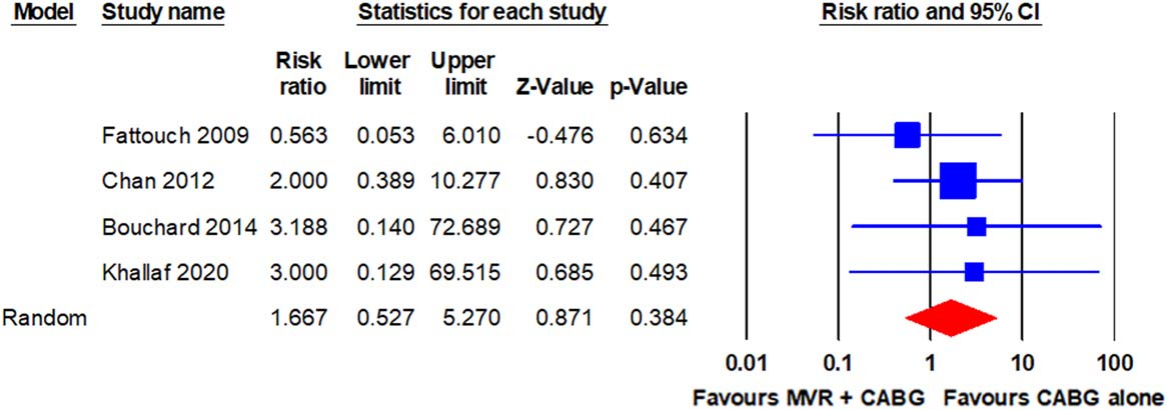
Forest plot for reoperation for bleeding or tamponade. CABG, coronary artery bypass grafting; MVR, mitral valve repair.

Reoperation for bleeding or tamponade was reported in four RCTs^9,21,22,24^. The pooled analysis using a random-effects model revealed no statistically significant difference in reoperation for bleeding or tamponade between patients who underwent MVR + CABG and those who underwent CABG alone (7/131 vs. 4/128; RR, 1.667; 95% CI, 0.527–5.270; *P* = 0.384). The analysis revealed no significant heterogeneity (I^2^ = 0%). The forest plot is shown in Fig. 7.

### Publication bias

Publication bias was assessed using funnel plots and Begg’s and Egger’s tests (Figures S3-S12, Pages 5-12, Supplemental Digital Content 4, http://links.lww.com/JS9/C150). The results revealed that no significant publication bias was observed for any of the pooled results. Detailed *P* values are presented in Table S3 (Page 8, Supplemental Digital Content 4, http://links.lww.com/JS9/C150).

### Sensitivity analysis

Regardless of whether the odds ratio was used as the summary statistic or each included study was excluded individually, the direction of the effect-estimate for operative mortality and long-term mortality remained unchanged (Figures S13-S16, Pages 13 and 14, Supplemental Digital Content 4, http://links.lww.com/JS9/C150). These findings indicate that the results are robust.

### Subgroup analysis

**Figure 8:**
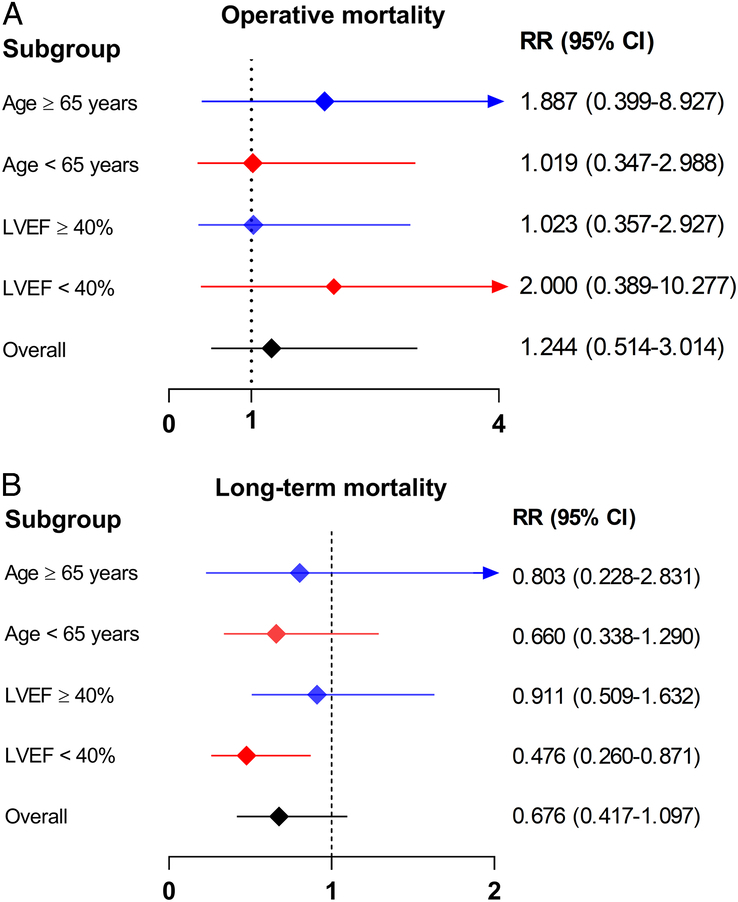
Subgroup analysis for the primary outcomes. The graph displays (A) operative mortality and (B) long-term mortality. LVEF, left ventricular ejection fraction; RR, risk ratio.

Subgroup analysis was performed to explore the effects of treatment strategies on operative and long-term mortalities among the different subgroups. The results showed no significant differences in operative mortality between patients who underwent MVR + CABG and those who underwent CABG alone in all subgroups. However, in terms of long-term mortality, patients who underwent MVR + CABG had better outcomes than those who underwent CABG alone in the subgroup of patients with LVEF less than 40% (RR, 0.476; 95% CI, 0.260–0.871). The subgroup analysis forest plot is shown in Fig. 8.

### Certainty of evidence

According to the GRADE scoring system (Figures S17 and S18, Pages15 and 16, Supplemental Digital Content 4, http://links.lww.com/JS9/C150), the quality of evidence was moderate in terms of operative mortality, long-term mortality, stroke, WRF, and reoperation for bleeding or tamponade. The quality of evidence for each outcome is shown in Table 2.

**Table 2:** GRADE evidence profile.

	Summary of findings			Quality assessment					
Outcomes	No. studies	No. patients	RR (95% CI)	Serious risk of bias	Serious inconsistency	Serious indirectness	Serious imprecision	Publication bias	Quality
Operative mortality	6	626	1.244 (0.514–3.014)	Not serious	Not serious	Serious^a^ (↓ 1 level)	Not serious	Unlikely	Moderate^b^
Long-term mortality	5	583	0.676 (0.417–1.097)	Not serious	Not serious	Serious^a^ (↓ 1 level)	Not serious	Unlikely	Moderate^b^
Stroke	4	484	2.425 (0.743–7.915)	Not serious	Not serious	Serious^a^ (↓ 1 level)	Not serious	Unlikely	Moderate^b^
WRF	3	479	1.257 (0.533–2.964)	Not serious	Not serious	Serious^a^ (↓ 1 level)	Not serious	Unlikely	Moderate^b^
Reoperation for bleeding or tamponade	4	259	1.667 (0.527–5.270)	Not serious	Not serious	Serious^a^ (↓ 1 level)	Not serious	Unlikely	Moderate^b^

GRADE, Grading of Recommendations, Assessment, Development and Evaluation; RR, risk ratio; WRF, worsening renal function.

a Total number of events is low.

b Moderate quality indicates further research is likely to have an important impact on our confidence in the estimate of effect and may change the estimate.

## Discussion

In this meta-analysis, we found that compared with CABG alone, MVR + CABG did not significantly increase the risk of operative mortality and postoperative stroke, WRF, and reoperation for bleeding or tamponade. However, no significant advantage was observed in terms of long-term mortality with MVR + CABG. The quality of the evidence for each outcome was rated as moderate. These findings provide the best available evidence on whether MVR should be performed during CABG in patients with moderate IMR.

In patients with coronary artery disease (CAD), particularly after MI, the presence and severity of MR have been identified as independent risk factors for mortality and cardiac function deterioration^4,27,28^. Currently, there is a general consensus that patients with moderate-to-severe or severe IMR should undergo combined CABG and mitral valve surgery^7,8^. However, there is conflicting evidence from various studies on the efficacy of MVR + CABG for the treatment of moderate IMR^29–32^, with some studies suggesting benefits, others indicating adverse effects, and some showing no significant effects. Currently, many surgeons favour a conservative approach, believing that good revascularization is sufficient to improve LV systolic and diastolic function, reverse remodelling, and ultimately decrease the IMR severity^33–35^. Furthermore, although MVR + CABG is associated with improved postoperative New York Heart Association (NYHA) functional classification and echocardiographic outcomes, it does not appear to confer long-term survival advantages. Our findings suggested that MVR + CABG did not reduce the risk of long-term mortality, although there was a trend toward reduction.

Compared with CABG alone, the addition of MVR to CABG surgery may necessitate the use of cardiopulmonary bypass or an extension of the bypass duration, potentially elevating the likelihood of death following surgery and the prevalence of subsequent complications. However, our findings indicated no significant difference in operative mortality between the two groups. Furthermore, our findings suggested that MVR + CABG did not lead to a higher incidence of common postoperative complications, such as stroke, WRF, and reoperation for bleeding or tamponade, compared with CABG alone. However, owing to insufficient data, we were unable to evaluate other clinical outcomes, such as low cardiac output syndrome, transfusion, and severe arrhythmias.

In the subgroup analysis, we found that MVR + CABG showed a significant long-term survival advantage compared with CABG alone among patients with LVEF less than 40%. This finding also explained the low heterogeneity of the pooled results. LVEF less than 40% indicates severe LV dysfunction, commonly suggesting that patients with CAD have extensive myocardial damage owing to past infarctions or chronic ischaemia. Consequently, performing CABG alone may have a negligible effect on improving cardiac function, and adverse remodelling of the LV is likely to persist. Concurrently, untreated IMR may accelerate this deterioration. This chain of events ultimately leads to heart failure and increased long-term mortality^36,37^.

In patients with moderate IMR, although good revascularization can alleviate the grade of postoperative MR, residual mild or greater regurgitation remains difficult to avoid. A recent study^38^ has reported that even mild MR can increase the risk of developing atrial fibrillation (AF) in the general population. However, no study has reported the long-term incidence of AF after CABG in patients with moderate IMR. Moreover, MVR + CABG can correct MR better than CABG alone. Therefore, theoretically, patients who undergo CABG + MVR would have a lower risk of developing AF and its long-term complications. Nevertheless, there is a lack of studies on this issue, and further exploration is required.

Several meta-analyses^39–43^ have assessed the therapeutic effects of MVR + CABG for patients with moderate IMR compared with CABG alone. Similar to our study, they found that MVR + CABG did not increase the risk of operative mortality but did not provide long-term survival benefits. Additionally, they observed a higher likelihood of moderate or severe MR recurrence and elevated NYHA functional class in patients who underwent CABG alone during follow-up. Nonetheless, these studies fall short in several respects compared with our study. Primarily, almost all the meta-analyses included observational studies, which significantly reduces the credibility of their findings. Moreover, the pooled results of different clinical research designs could not be evaluated for quality of evidence. Lastly, they overlooked two RCTs, and there was considerable heterogeneity in some of the pooled results.

Although we enhanced the robustness of our evidence by exclusively incorporating RCTs into the present meta-analysis, our study had some limitations. First, the sample sizes of included RCTs were relatively small, with only two trials comprising greater than 100 participants. This could have affected the reliability of the results. Second, owing to the nature of the surgical interventions, it was challenging to achieve blinding for both the investigators and participants in the included studies. This may have introduced an unpredictable bias into the results. Third, although we conducted Begg’s and Egger’s tests to assess publication bias for each outcome, bias may still exist owing to the limited number of RCTs included. Finally, the generalizability of the evidence derived from RCTs may be limited in real-world scenarios because of the strict inclusion and exclusion criteria.

## Conclusion

In summary, the findings of this meta-analysis suggest that adding MVR to CABG does not confer improved clinical outcomes compared with CABG alone in patients with moderate IMR. However, correction of moderate IMR in patients with severe LV dysfunction (LVEF < 40%) at the time of CABG may improve long-term survival.

## Ethical approval

This study does not require.

## Consent

This study does not require.

## Source of funding

This study was funded by major scientific and technological innovation research and development project of Beijing Anzhen Hospital affiliated to Capital Medical University and high-end foreign expert introduction plan (grant number: G2022001039L).

## Author contribution

H.Z. and Y.L. conceived and designed the study. X.L. and B.H. performed literature search, data collection and data analysis. X.L. wrote the manuscript. X.L. and W.J. examined and corrected the manuscript. All authors agreed with the results and conclusions of the manuscript.

## Conflicts of interest disclosure

None declared.

## Research registration unique identifying number (UIN)

This study was registered with PROSPERO (ID: CRD42023470927).

## Guarantor

Hongjia Zhang.

## Data statement

The data generated during the current study are available from the corresponding author upon reasonable request.

## Provenance and peer review

Our paper was not invited.

## Supplementary Material
