# Supplementary material for: Efficacy of mitral valve repair in combination with coronary revascularization for moderate ischaemic mitral regurgitation: a systematic review and meta-analysis of randomized controlled trials
Source: Int J Surg. 2024 Mar 19;110(6):3879–87. doi: 10.1097/JS9.0000000000001277 (PMC11175805; doi:10.1097/JS9.0000000000001277)
Supplement: Supplementary file 2 [file js9-110-3879-s002.doc]

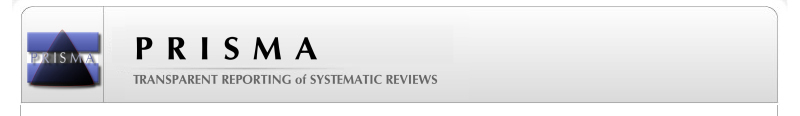
**PRISMA 2009 Flow Diagram**

aa

**Screening**

**Included**

**Eligibility**

**Identification**

Records identified through database searching (n = 2119)

Additional records identified through other sources (n = 0)

Records after duplicates removed (n = 1017)

Records screened (n = 1017)

Records excluded (n = 1003)

Full-text of RCTs assessed for eligibility (n = 14)

Full-text articles excluded (n = 8)

- Mixed MR etiologies (n = 5)
- Non-moderate IMR (n = 2)
- MVR + CABG vs. CABG + Coapsys device (n = 1)

RCTs included in meta-analysis (n = 6)
