# Supplementary material for: Efficacy of mitral valve repair in combination with coronary revascularization for moderate ischaemic mitral regurgitation: a systematic review and meta-analysis of randomized controlled trials
Source: Int J Surg. 2024 Mar 19;110(6):3879–87. doi: 10.1097/JS9.0000000000001277 (PMC11175805; doi:10.1097/JS9.0000000000001277)
Supplement: Supplementary file 3 [file js9-110-3879-s003.doc]

**AMSTAR 2**

**1. Did the research questions and inclusion criteria for the review include the components of PICO?**

For Yes:

Optional (recommended)


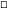
 Timeframe for follow-up


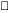
 Population

Yes

No

√
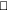


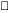
 Intervention


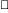
 Comparator group


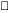
 Outcome

**2. Did the report of the review contain an explicit statement that the review methods were established prior to the conduct of the review and did the report justify any significant deviations from the protocol?**

For Partial Yes: For Yes:

The authors state that they had a written As for partial yes, plus the protocol

protocol or guide that included ALL the should be registered and should also

following: have specified:

√ Yes


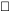
 review question(s)
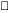
 a meta-analysis/synthesis
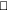
 Partial Yes


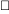
 a search strategy plan, if appropriate, *and*
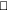
No


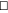
 inclusion/exclusion criteria
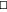
 a plan for investigating


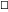
 a risk of bias assessment causes of heterogeneity


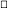
 justification for any

deviations from the protocol

**3. Did the review authors explain their selection of the study designs for inclusion in the review?**

For Yes, the review should satisfy ONE of the following:


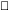
 *Explanation for* including only RCTs √ Yes


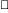
 OR *Explanation for* including only NRSI
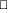
 No


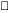
 OR *Explanation for* including both RCTs and NRSI

**4. Did the review authors use a comprehensive literature search strategy?**

For Partial Yes (all the following): For Yes, should also have (all the

following):


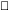
 searched at least 2 databases
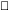
 searched the reference √ Yes

(relevant to research question) lists/bibliographies of
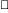
 Partial Yes


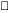
 provided keyword and/or included studies
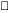
 No

search strategy
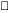
 searched trial/study


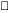
 justified publication registries

restrictions (eg, language)
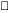
 included/consulted content

experts in the field


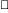
 where relevant, searched for

grey literature


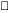
 conducted search within 24

months of completion of the

review

**5. Did the review authors perform study selection in duplicate?**

For Yes, either ONE of the following:


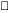
 at least two reviewers independently agreed on selection of eligible √ Yes

studies and achieved consensus on which studies to include
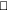
 No


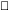
 OR two reviewers selected a sample of eligible studies and achieved

good agreement (at least 80 per cent), with the remainder selected by

one reviewer

**6. Did the review authors perform data extraction in duplicate?**

For Yes, either ONE of the following:


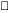
 at least two reviewers achieved consensus on which data to extract √ Yes

from included studies


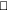
 No


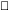
 OR two reviewers extracted data from a sample of eligible studies and

achieved good agreement (at least 80 per cent), with the remainder

extracted by one reviewer

**7. Did the review authors provide a list of excluded studies and justify the exclusions?**

For Yes, must also have:

For Partial Yes:


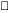
 Justified the exclusion from


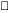
 provided a list of all

potentially relevant studies

that were read in full text form but excluded from the review

Yes

Partial Yes

No

√
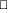

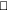


the review of each

potentially relevant study

**8. Did the review authors describe the included studies in adequate detail?**

For Partial Yes (ALL the following): For Yes, should also have ALL the

following:


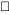
 described populations
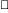
 described population in √ Yes


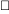
 described interventions detail
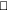
 Partial Yes


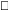
 described comparators
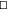
 described intervention and
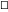
 No

comparator in detail


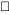
 described outcomes


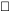
 described research designs relevant)

(including doses where


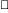
 described study’s setting


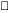
 timeframe for follow-up

**9. Did the review authors use a satisfactory technique for assessing the risk of bias (RoB) in individual studies that were included in the review?**

**RCTs**

For Yes, must also have assessed

For Partial Yes, must have assessed RoB from

RoB from:


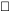
 unconcealed allocation, *and*


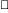
 allocation sequence that was not truly random, *and*


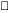
 selection of the reported

result from among multiple measurements or analyses of a specified outcome

For Yes, must also have assessed RoB:


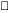
 methods used to ascertain exposures and outcomes,

√ Yes


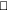
 Partial Yes
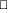
 No


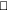
 Includes only NRSI


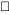
 lack of blinding of patients

and assessors when assessing

outcomes (unnecessary for

objective outcomes such as all

cause mortality)

**NRSI**

For Partial Yes, must have assessed


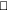
 Yes


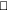
 Partial Yes
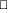
 No

√ Includes only RCTs

RoB:


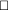
 from confounding, *and*


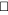
 from selection bias

*and*


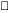
 selection of the reported

result from among multiple

measurements or analyses of

a specified outcome

**10. Did the review authors report on the sources of funding for the studies included in the review?**

For Yes


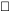
 Must have reported on the sources of funding for individual studies included

Yes

No

√
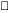


in the review. Note: Reporting that the reviewers looked for this information

but it was not reported by study authors also qualifies

**11. If meta-analysis was performed did the review authors use appropriate methods for statistical combination of results?**

**RCTs**

For Yes:

√ Yes
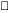
 No


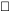
 The authors justified combining the data in a meta-analysis


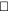
 AND they used an appropriate weighted technique to combine

study results and adjusted for heterogeneity if present


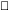
 No meta-analysis


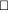
 AND investigated the causes of any heterogeneity

conducted

**For NRSI**

For Yes:


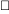
 The authors justified combining the data in a meta-analysis

√ Yes


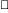
 No


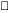
 No meta-analysis conducted


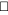
 AND they used an appropriate weighted technique to combine

study results, adjusting for heterogeneity if present


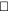
 AND they statistically combined effect estimates from NRSI

that were adjusted for confounding, rather than combining

raw data, or justified combining raw data when adjusted effect

estimates were not available


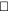
 AND they reported separate summary estimates for RCTs and

NRSI separately when both were included in the review

**12. If meta-analysis was performed, did the review authors assess the potential impact of RoB in individual studies on the results of the meta-analysis or other evidence synthesis?**

For Yes:


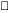
 included only low risk of bias RCTs

√ Yes


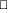
 No


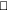
 No meta-analysis conducted


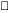
 OR, if the pooled estimate was based on RCTs and/or NRSI at variable

RoB, the authors performed analyses to investigate possible impact of

RoB on summary estimates of effect

**13. Did the review authors account for RoB in individual studies when interpreting/discussing the results of the review?**

For Yes:


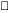
 included only low risk of bias RCTs

Yes

No

√
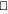


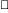
 OR, if RCTs with moderate or high RoB, or NRSI were included the

review provided a discussion of the likely impact of RoB on the results

**14. Did the review authors provide a satisfactory explanation for, and discussion of, any heterogeneity observed in the results of the review?**

For Yes:


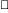
 There was no significant heterogeneity in the results


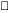
 OR if heterogeneity was present the authors performed an investigation

Yes

No

√
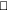


of sources of any heterogeneity in the results and discussed the impact

of this on the results of the review

**15. If they performed quantitative synthesis did the review authors carry out an adequate**

**investigation of publication bias (small study bias) and discuss its likely impact on the results of the review?**

For Yes:


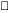
 performed graphical or statistical tests for publication bias and √ Yes

discussed the likelihood and magnitude of impact of publication bias
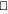
 No


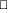
 No meta-analysis conducted

**16. Did the review authors report any potential sources of conflict of interest, including any funding they received for conducting the review?**

For Yes:


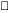
 The authors reported no competing interests OR

Yes

No

√
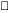


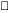
 The authors described their funding sources and how they

managed potential conflicts of interest
