# Supplementary material for: Efficacy of mitral valve repair in combination with coronary revascularization for moderate ischaemic mitral regurgitation: a systematic review and meta-analysis of randomized controlled trials
Source: Int J Surg. 2024 Mar 19;110(6):3879–87. doi: 10.1097/JS9.0000000000001277 (PMC11175805; doi:10.1097/JS9.0000000000001277)
Supplement: Supplementary file 4 [file js9-110-3879-s004.pdf]

Efficacy of mitral valve repair in combination with coronary revascularization for moderate ischemic mitral regurgitation: a systematic review and meta-analysis of randomized controlled trials

Supplemental data

Content

Search results......2

Risk of bias assessment......4

Publication bias assessment......5

Sensitivity analysis......13

GRADE assessment......15

Search results

Table S1. Details of literature searching		
Strategy	Databases	Final date of searching
(Ischemic OR functional OR secondary) AND (mitral regurgitation OR mitral incompetence OR mitral insufficiency OR mitral dysfunction) AND (randomized OR clinical trials)	PubMed, Web of Science, and Cochrane Library	Oct. 5, 2023

Tabe S2. Inclusion and exclusion of studies	
Included studies [1-6]	Excluded studies [7-14]
	Reasons for exclusion<br>Patients had mixed MR etiologies [7, 8, 12, 13, 14]<br>The grade of IMR was severe [9, 11]<br>comparison was MVR + CABG vs. CABG + LV reshaping [10]

Potential eligible studies[1-14]

1. Bouchard D, Jensen H, Carrier M, et al. Effect of systematic downsizing rigid ring annuloplasty in patients with moderate ischemic mitral regurgitation. J Thorac Cardiovasc Surg. 2014;147:1471-7.
2. Chan KM, Punjabi PP, Flather M, et al. Coronary artery bypass surgery with or without mitral valve annuloplasty in moderate functional ischemic mitral regurgitation: final results of the Randomized Ischemic Mitral Evaluation (RIME) trial. Circulation. 2012;126:2502-10.
3. Fattouch K, Guccione F, Sampognaro R, et al. POINT: Efficacy of adding mitral valve restrictive annuloplasty to coronary artery bypass grafting in patients with moderate ischemic mitral valve regurgitation: a randomized trial. J Thorac Cardiovasc Surg. 2009;138:278-85.
4. Kareva YE, Efendiev VU, Rakhmonov SS, et al. [Long-Term Survival of Patients with Ischemic Heart Disease After Surgical Correction of Moderate Ischemic Mitral Regurgitation]. Kardiologiia. 2019;59:13-19.
5. Khallaf A, Elzayadi M, Alkady H, et al. Results of Coronary Artery Bypass Grafting Alone Versus Combined Surgical Revascularization and Mitral Repair In Patients with Moderate Ischemic Mitral Regurgitation. Heart Surg Forum. 2020;23:E270-E275.
6. Smith PK, Puskas JD, Ascheim DD, et al. Surgical treatment of moderate ischemic mitral regurgitation. N Engl J Med. 2014;371:2178-88.
7. Feldman T, Foster E, Glower DD, et al. Percutaneous repair or surgery for mitral

regurgitation. N Engl J Med. 2011;364:1395-406.
8. Acker MA, Bolling S, Shemin R, et al. Mitral valve surgery in heart failure: insights from the Acorn Clinical Trial. J Thorac Cardiovasc Surg. 2006;132:568-77, 577.e1-4.
9. Acker MA, Parides MK, Perrault LP, et al. Mitral-valve repair versus replacement for severe ischemic mitral regurgitation. N Engl J Med. 2014;370:23-32.
10. Grossi EA, Patel N, Woo YJ, et al. Outcomes of the RESTOR-MV Trial (Randomized Evaluation of a Surgical Treatment for Off-Pump Repair of the Mitral Valve). J Am Coll Cardiol. 2010;56:1984-93.
11. Nappi F, Lusini M, Spadaccio C, et al. Papillary Muscle Approximation Versus Restrictive Annuloplasty Alone for Severe Ischemic Mitral Regurgitation. J Am Coll Cardiol. 2016;67:2334-2346.
12. Obadia JF, Messika-Zeitoun D, Leurent G, et al. Percutaneous Repair or Medical Treatment for Secondary Mitral Regurgitation. N Engl J Med. 2018;379:2297-2306.
13. Stone GW, Lindenfeld J, Abraham WT, et al. Transcatheter Mitral-Valve Repair in Patients with Heart Failure. N Engl J Med. 2018;379:2307-2318.
14. Witte KK, Lipiecki J, Siminiak T, et al. The REDUCE FMR Trial: A Randomized Sham-Controlled Study of Percutaneous Mitral Annuloplasty in Functional Mitral Regurgitation. JACC Heart Fail. 2019;7:945-955.

Risk of bias assessment (ROB 2 Tool)

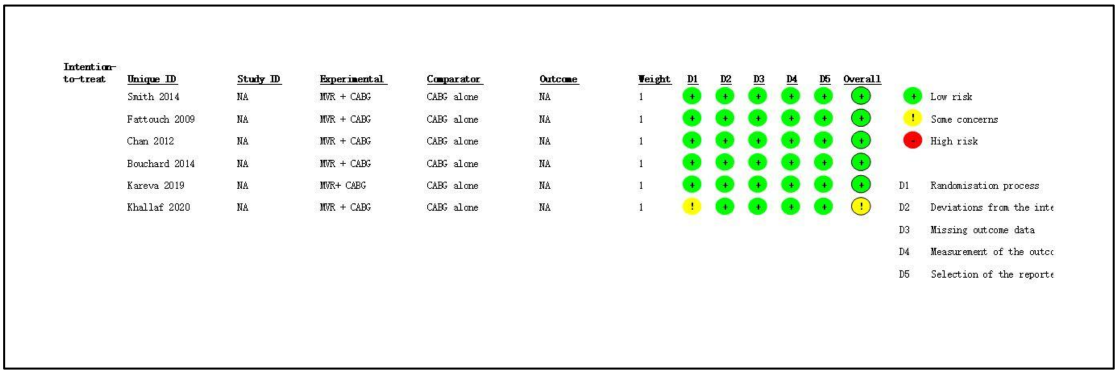

Figure S1. Original figure of ROB assessment. Distribution of the bias for each study.

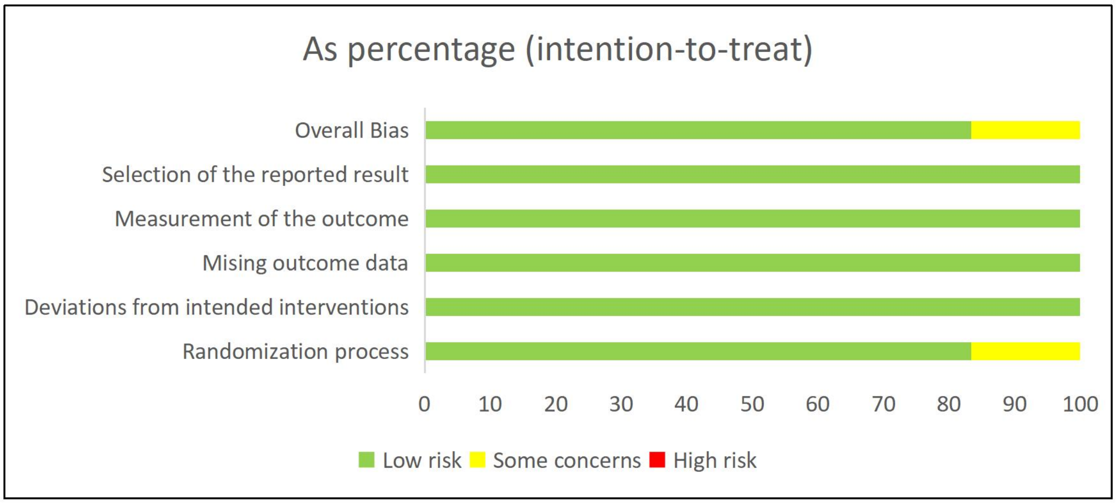

Figure S2. Original figure of ROB assessment. Summary of the risk of bias

Publication bias assessment

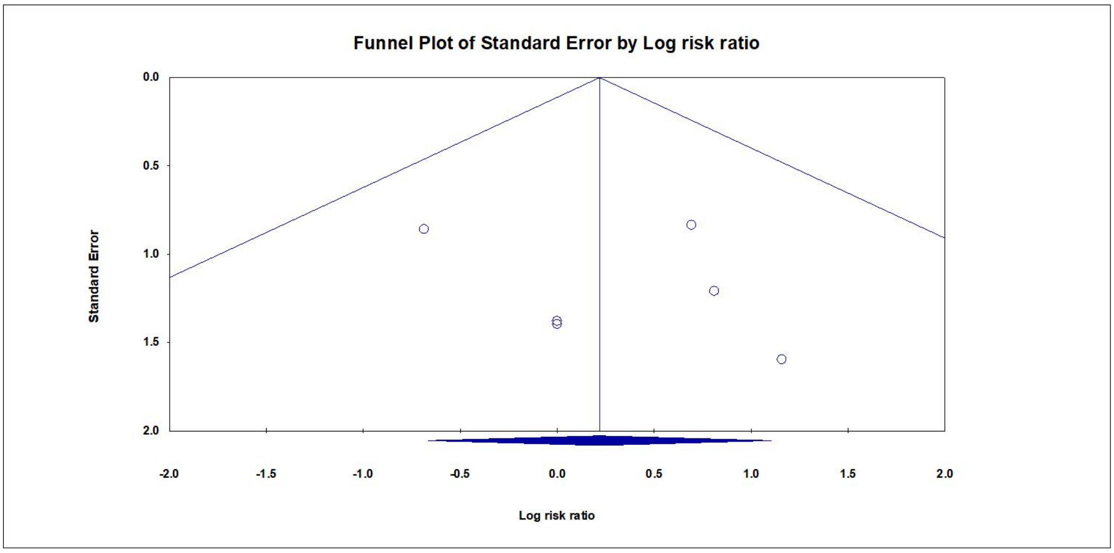

Figure S3. Funnel plot for operative mortality.

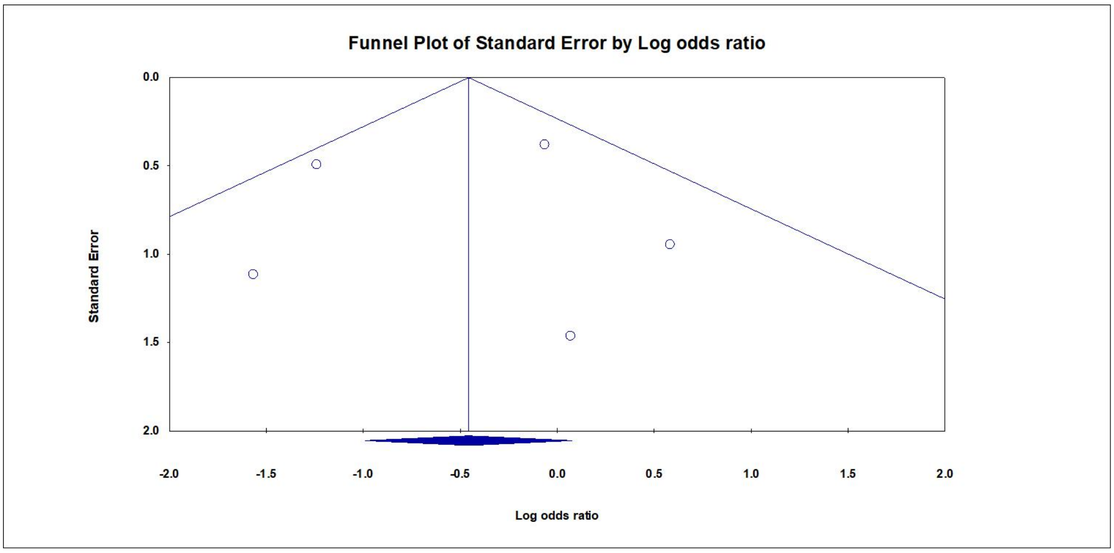

Figure S4. Funnel plot for long-term mortality.

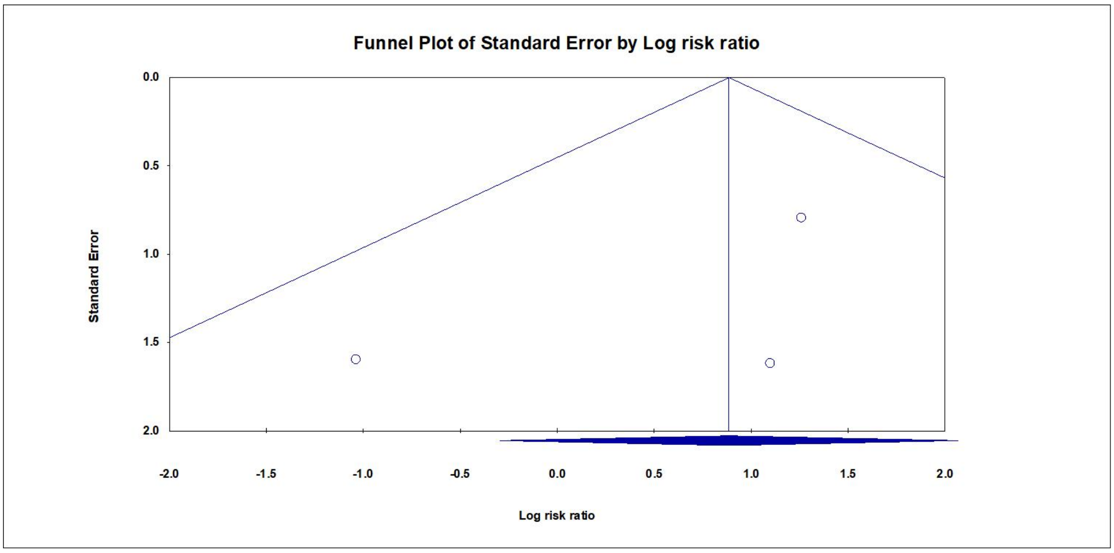

Figure S5. Funnel plot for stroke.

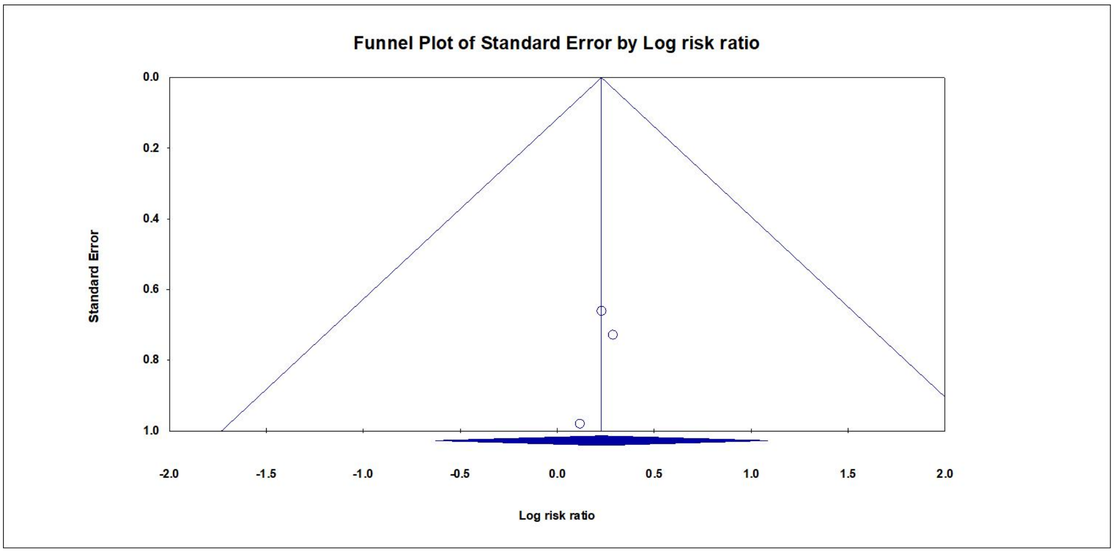

Figure S6. Funnel plot for worsening renal function.

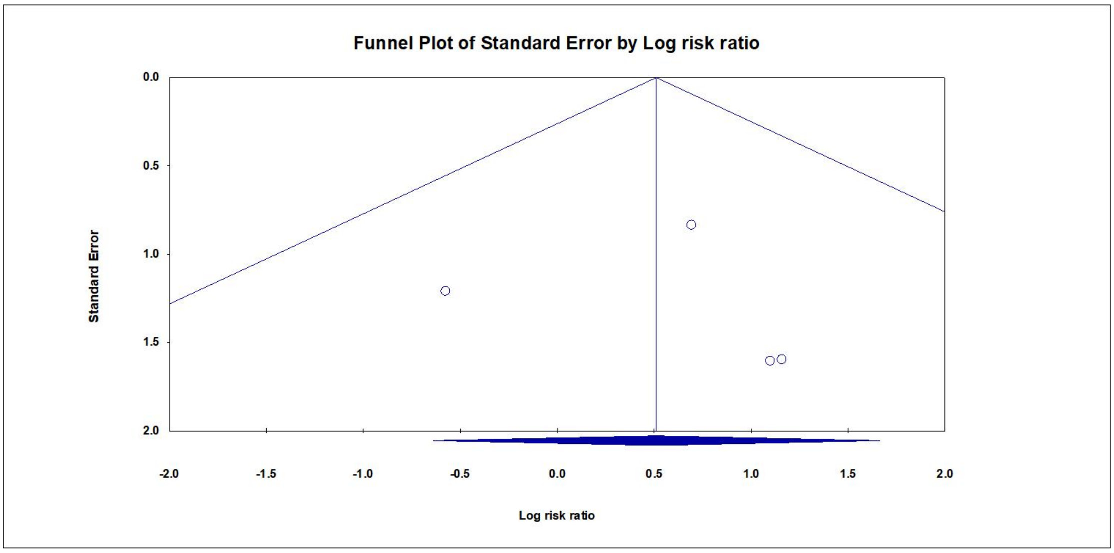

Figure S7. Funnel plot for reoperation for bleeding or tamponade.

Table S3. Quantitative assessment of publication bias for each outcome					
Method	Operative mortality	Long-term mortality	Stroke	WRF	Reoperation for bleeding or tamponade
Begg's test	0.260	0.806	1.000	1.000	0.734
Egger's test	0.223	0.669	0.364	0.397	0.419
WRF: worsening renal function. All values indicate the P-values.					

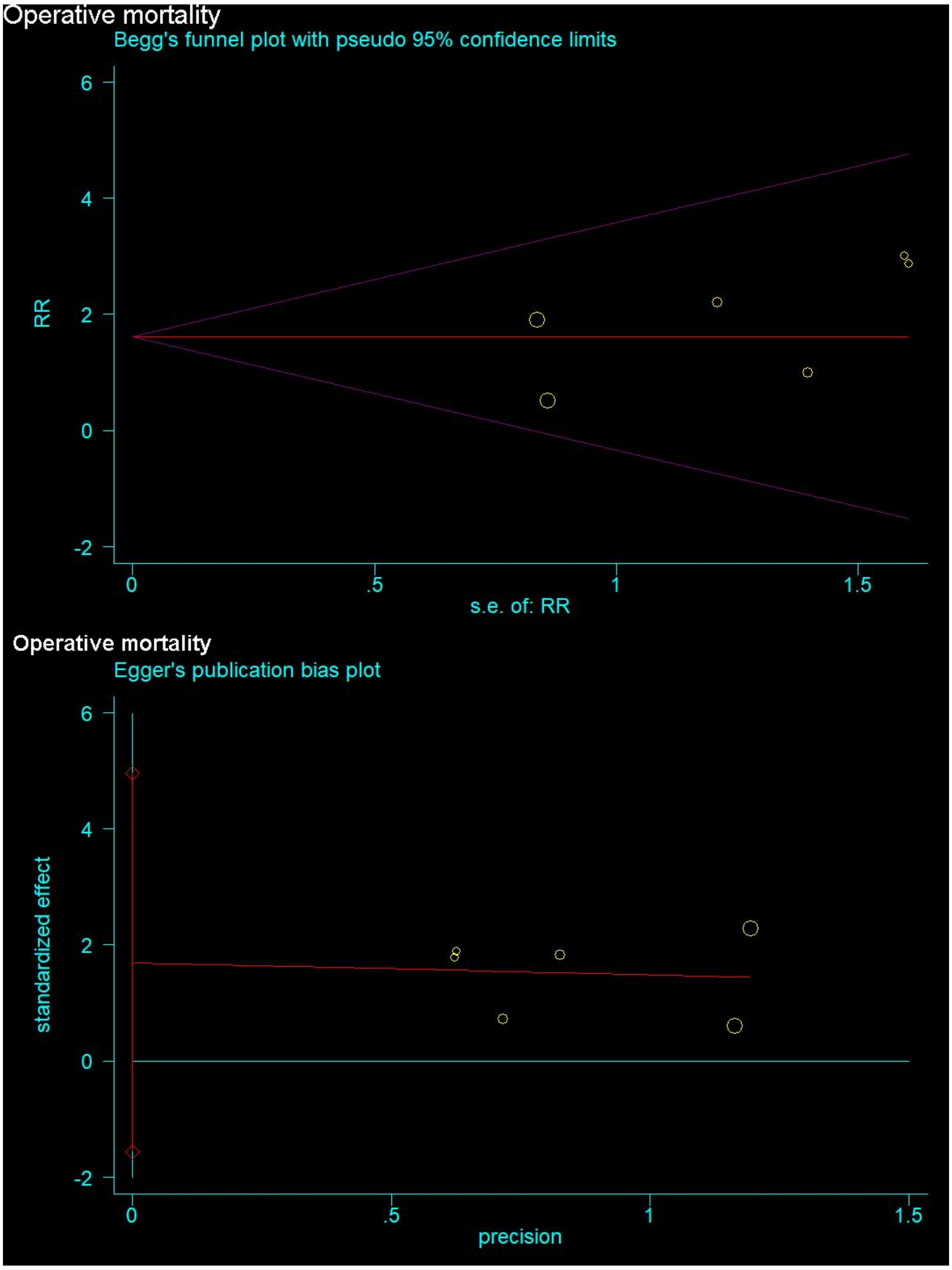

Figure S8. Plots of Begg's and Egger's tests for operative mortality.

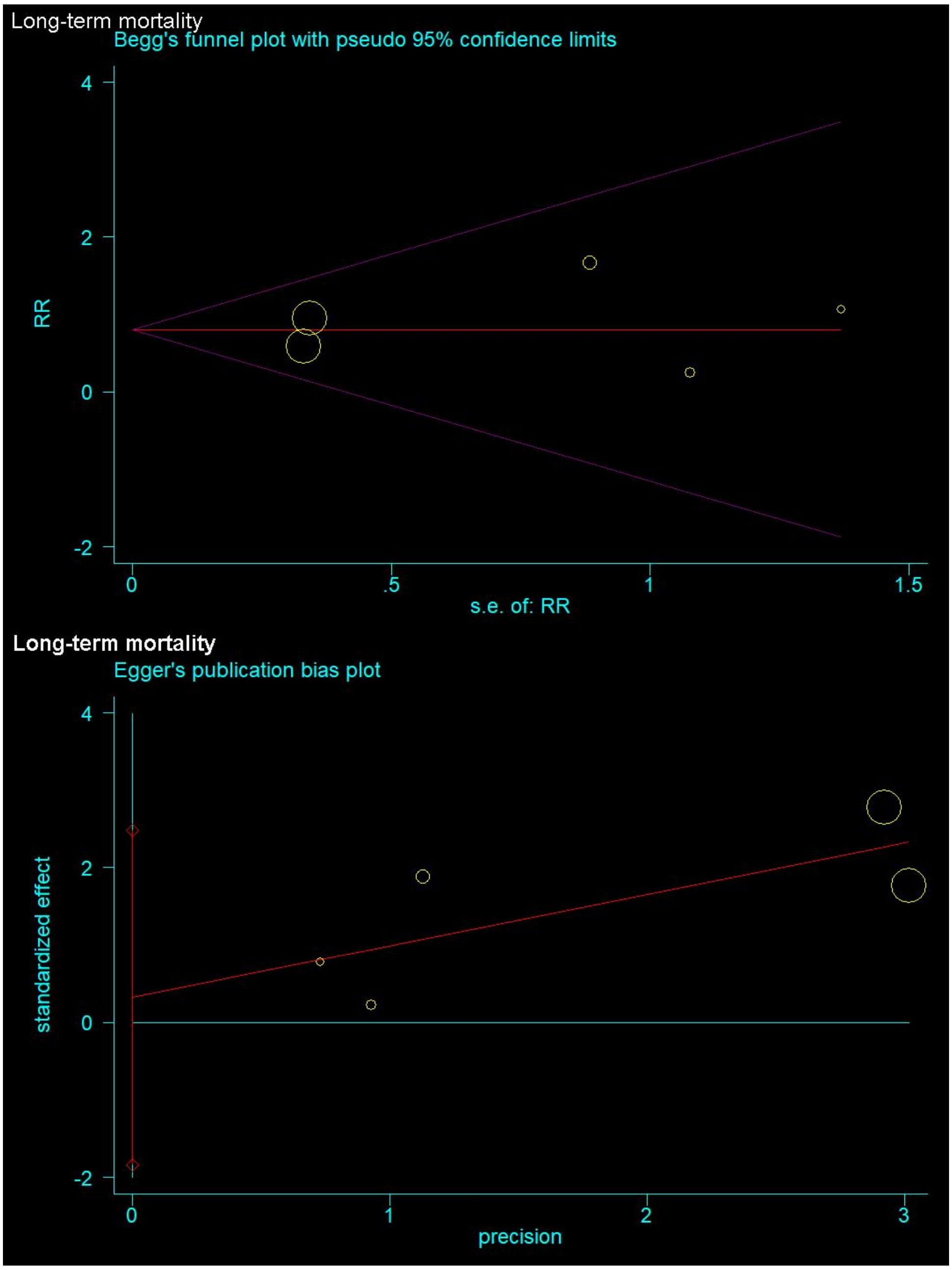

Figure S9. Plots of Begg's and Egger's tests for long-term mortality.

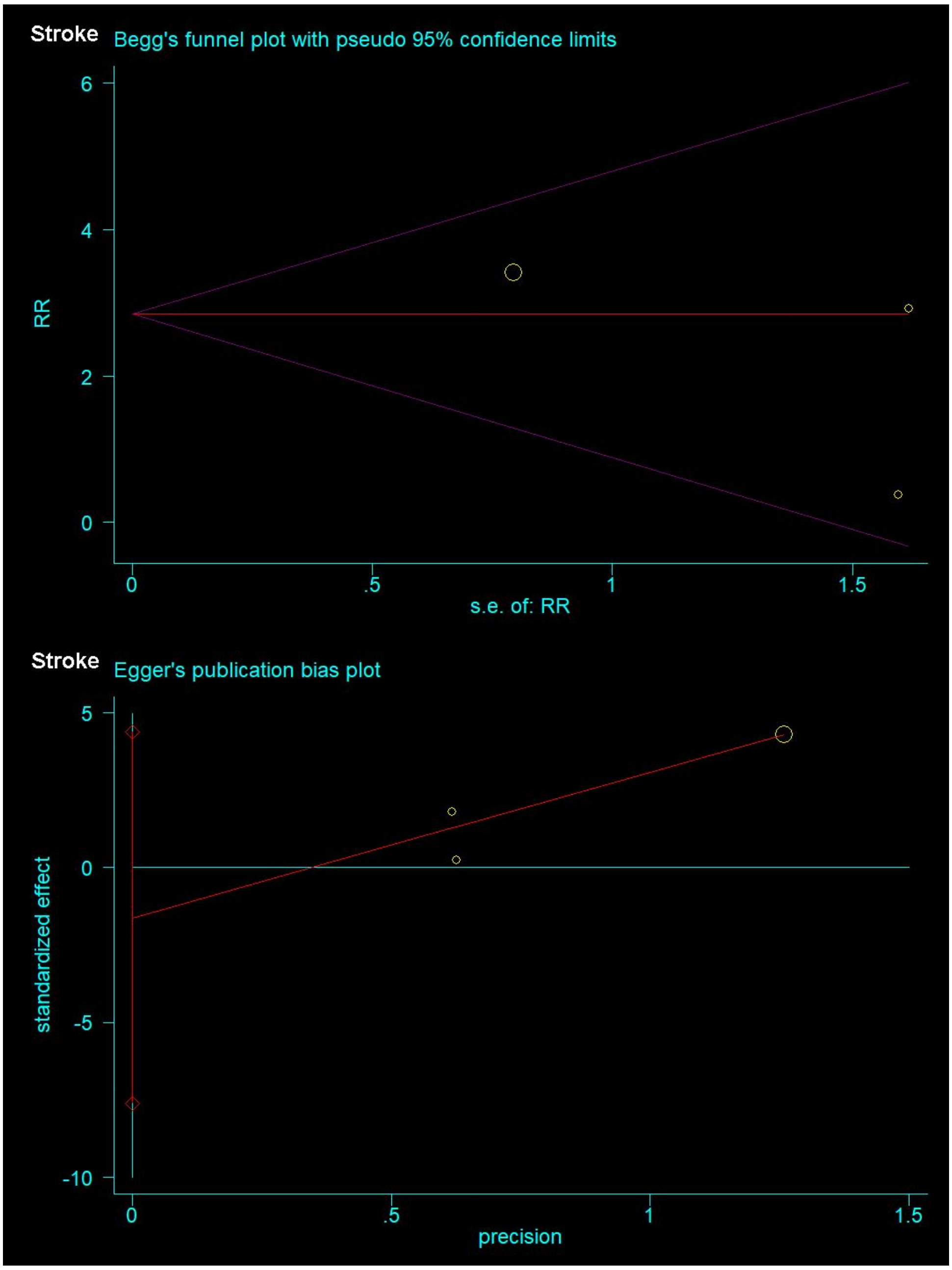

Figure S10. Plots of Begg’s and Egger’s tests for stroke.

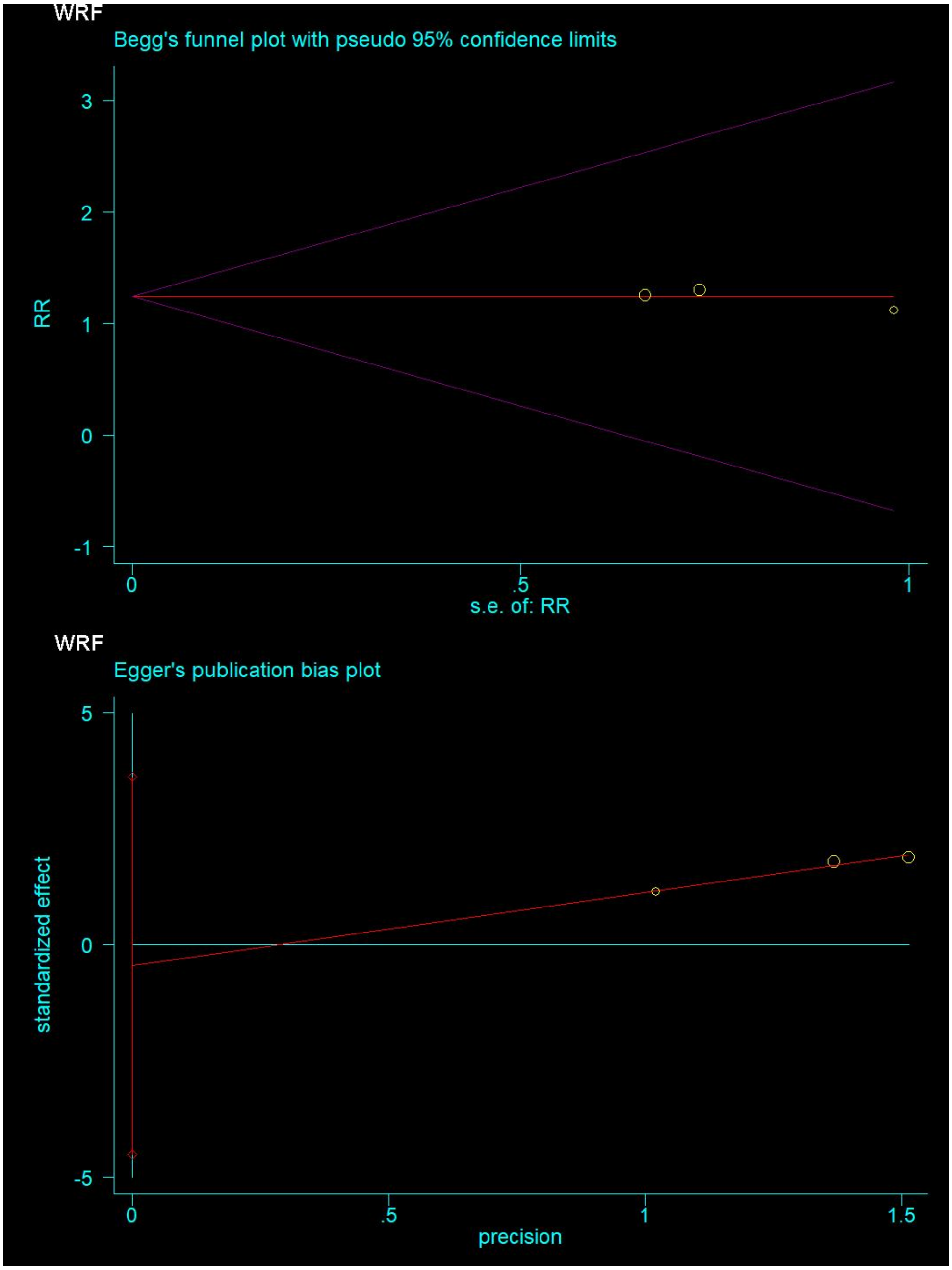

Figure S11. Plots of Begg's and Egger's tests for worsening renal function.

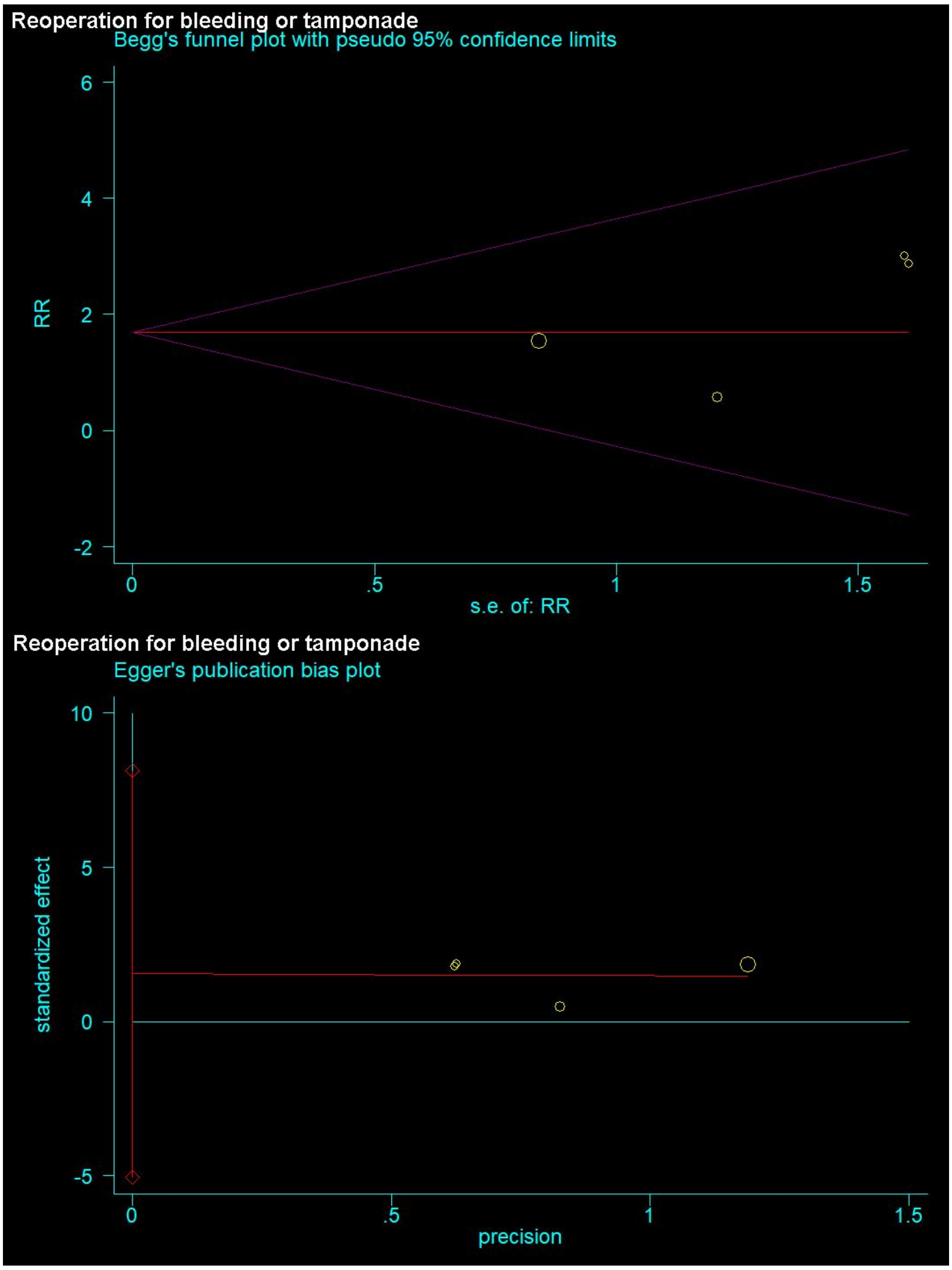

Figure S12. Plots of Begg's and Egger's tests for reoperation for bleeding or tamponade.

Sensitivity

analysis

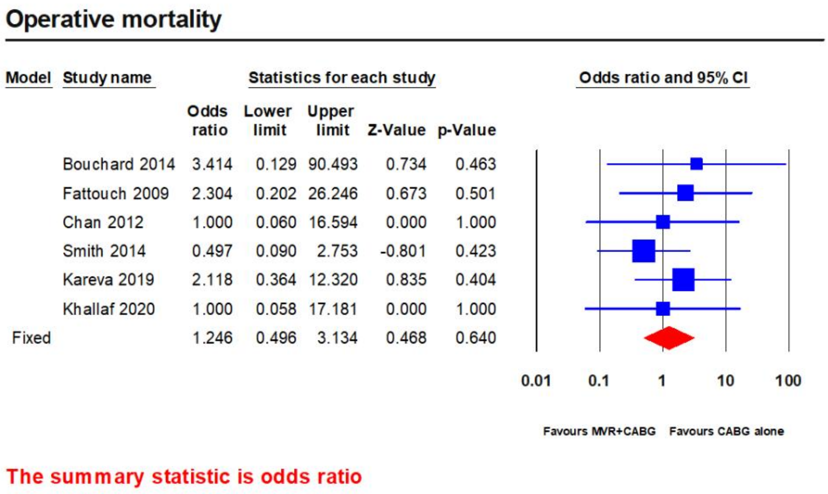

Figure S13. Plot of sensitivity analysis for operative mortality. The summary statistic is odds Ratio

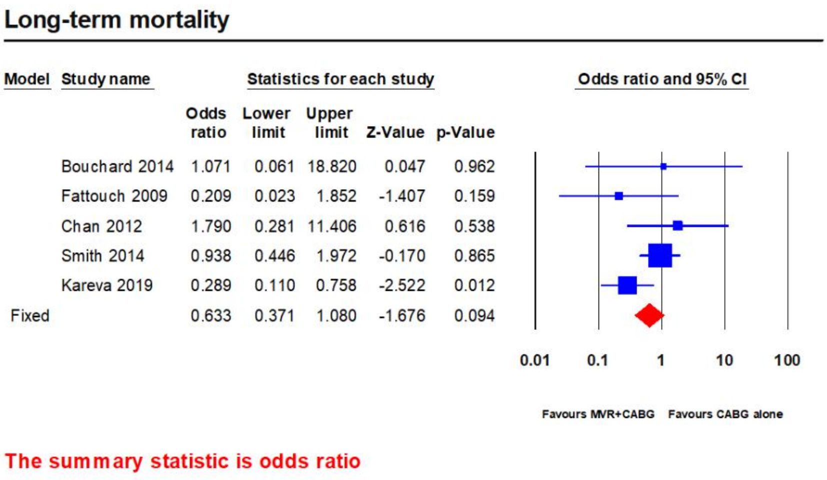

Figure S14. Plot of sensitivity analysis for long-term mortality. The summary statistic is odds ratio.

Operative mortality

Study name	Point	Lower limit	Upper limit	Z-Value	p-Value
	Statistics with study removed				
Bouchard 2014	1.146	0.456	2.884	0.290	0.771
Fattouch 2009	1.130	0.436	2.933	0.252	0.801
Chan 2012	1.276	0.501	3.251	0.512	0.609
Smith 2014	1.758	0.622	4.975	1.064	0.287
Kareva 2019	1.023	0.357	2.927	0.042	0.966
Khallaf 2020	1.277	0.501	3.258	0.512	0.608
	1.244	0.514	3.014	0.484	0.628

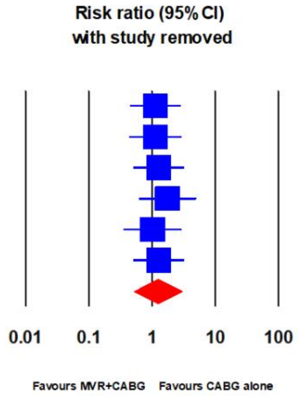

Excluding each included study one by one

Figure S15. Plot of sensitivity analysis for operative mortality. Excluding each included study individually.

Long-term mortality

Study name	Point	Lower limit	Upper limit	Z-Value	p-Value
	Statistics with study removed				
Bouchard 2014	0.659	0.431	1.007	-1.928	0.054
Fattouch 2009	0.697	0.454	1.069	-1.655	0.098
Chan 2012	0.628	0.408	0.968	-2.109	0.035
Smith 2014	0.531	0.310	0.910	-2.301	0.021
Kareva 2019	0.911	0.509	1.632	-0.313	0.754
	0.666	0.438	1.014	-1.897	0.058

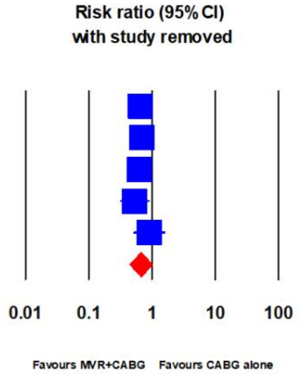

Excluding each included study one by one

Figure S16. Plot of sensitivity analysis for long-term mortality. Excluding each included study individually.

GRADE assessment

Summary of findings:

MVR + CABG compared to CABG alone for moderate IMR

Patient or population: moderate IMR
Setting:
Intervention: MVR + CABG
Comparison: CABG alone

Outcome<br>№ of participants<br>(studies)	Relative effect<br>(95% CI)	Anticipated absolute effects (95% CI)			Certainty	What happens
				**Difference**		
Operative mortality<br>№ of participants: 626<br>(6 RCTs)	**RR 1.37**<br>(0.56 to 3.36)	**Study population**			⊕⊕⊕◯<br>Moderate[a]	
		2.5%	**3.5%**<br>(1.4 to 8.5)	**0.9% more**<br>(1.1 fewer to 6 more)		
		**Moderate**				
		2.2%	**3.0%**<br>(1.2 to 7.4)	**0.8% more**<br>(1 fewer to 5.2 more)		
Long-term mortality<br>№ of participants: 583<br>(5 RCTs)	**RR 0.68**<br>(0.42 to 1.10)	**Study population**			⊕⊕⊕◯<br>Moderate[a]	
		15.1%	**10.3%**<br>(6.3 to 16.6)	**4.8% fewer**<br>(8.8 fewer to 1.5 more)		
		**Moderate**				
		9.3%	**6.3%**<br>(3.9 to 10.2)	**3.0% fewer**<br>(5.4 fewer to 0.9 more)		
Stroke<br>№ of participants: 484<br>(4 RCTs)	**RR 2.43**<br>(0.74 to 7.91)	**Study population**			⊕⊕⊕◯<br>Moderate[a]	
		1.2%	**3.0%**<br>(0.9 to 9.8)	**1.8% more**<br>(0.3 fewer to 8.5 more)		
		**Moderate**				
		0.7%	**1.7%**<br>(0.5 to 5.5)	**1.0% more**<br>(0.2 fewer to 4.8 more)		
Worsening renal function<br>№ of participants: 479<br>(3 RCTs)	**RR 1.26**<br>(0.53 to 2.96)	**Study population**			⊕⊕⊕◯<br>Moderate[a]	
		3.7%	**4.7%**<br>(2 to 11)	**1.0% more**<br>(1.7 fewer to 7.3 more)		
		**Moderate**				
		3.7%	**4.7%**<br>(2 to 11)	**1.0% more**<br>(1.7 fewer to 7.3 more)		
Reoperation for bleeding or tamponade<br>№ of participants: 249<br>(4 RCTs)	**RR 1.67**<br>(0.53 to 5.27)	**Study population**			⊕⊕⊕◯<br>Moderate[a]	
		3.1%	**5.2%**<br>(1.7 to 16.5)	**2.1% more**<br>(1.5 fewer to 13.3 more)		
		**Moderate**				
		1.9%	**3.2%**<br>(1 to 10)	**1.3% more**<br>(0.9 fewer to 8.1 more)		

*__The risk in the intervention group__ (and its 95% confidence interval) is based on the assumed risk in the comparison group and the **relative effect** of the intervention (and its 95% CI).

CI: confidence interval; **RR:** risk ratio

GRADE Working Group grades of evidence
High certainty: we are very confident that the true effect lies close to that of the estimate of the effect.
Moderate certainty: we are moderately confident in the effect estimate: the true effect is likely to be close to the estimate of the effect, but there is a possibility that it is substantially different.
Low certainty: our confidence in the effect estimate is limited: the true effect may be substantially different from the estimate of the effect.
Very low certainty: we have very little confidence in the effect estimate: the true effect is likely to be substantially different from the estimate of effect.

Explanations

a. Total number of events is low.

Figure S17. Summary of findings table.

MVR + CABG compared to CABG alone for moderate IMR
Bibliography: MVR + CABG vs CABG alone for moderate IMR.

Certainty assessment							Summary of findings				
Participants (studies) Follow-up	Risk of bias	Inconsistency	Indirectness	Imprecision	Publication bias	Overall certainty of evidence	Study event rates (%) With CABG alone	Study event rates (%) With MVR + CABG	Relative effect (95% CI)	Anticipated absolute effects: Risk with CABG alone	Anticipated absolute effects: Risk difference with MVR + CABG
**Operative mortality**											
626 (6 RCTs)	not serious	not serious	not serious	serious[a]	none	⊕⊕⊕◯ Moderate	8/317 (2.5%)	11/309 (3.6%)	**RR 1.37** (0.56 to 3.36)	**Study population**	
										25 per 1,000	**9 more per 1,000** (from 11 fewer to 60 more)
										**Moderate**	
										22 per 1,000	**8 more per 1,000** (from 10 fewer to 52 more)
**Long-term mortality**											
583 (5 RCTs)	not serious	not serious	not serious	serious[a]	none	⊕⊕⊕◯ Moderate	45/298 (15.1%)	30/285 (10.5%)	**RR 0.68** (0.42 to 1.10)	**Study population**	
										151 per 1,000	**48 fewer per 1,000** (from 88 fewer to 15 more)
										**Moderate**	
										93 per 1,000	**30 fewer per 1,000** (from 54 fewer to 9 more)
**Stroke**											
484 (4 RCTs)	not serious	not serious	not serious	serious[a]	none	⊕⊕⊕◯ Moderate	3/243 (1.2%)	9/241 (3.7%)	**RR 2.43** (0.74 to 7.91)	**Study population**	
										12 per 1,000	**18 more per 1,000** (from 3 fewer to 85 more)
										**Moderate**	
										7 per 1,000	**10 more per 1,000** (from 2 fewer to 48 more)
**Worsening renal function**											
479 (3 RCTs)	not serious	not serious	not serious	serious[a]	none	⊕⊕⊕◯ Moderate	9/243 (3.7%)	11/236 (4.7%)	**RR 1.26** (0.53 to 2.96)	**Study population**	
										37 per 1,000	**10 more per 1,000** (from 17 fewer to 73 more)
										**Moderate**	
										37 per 1,000	**10 more per 1,000** (from 17 fewer to 73 more)
**Reoperation for bleeding or tamponade**											
249 (4 RCTs)	not serious	not serious	not serious	serious[a]	none	⊕⊕⊕◯ Moderate	4/128 (3.1%)	7/121 (5.8%)	**RR 1.67** (0.53 to 5.27)	**Study population**	
										31 per 1,000	**21 more per 1,000** (from 15 fewer to 133 more)
										**Moderate**	
										19 per 1,000	**13 more per 1,000** (from 9 fewer to 81 more)

CI: confidence interval; **RR:** risk ratio

Explanations

a. Total number of events is low.

Figure S18. GRADE evidence profile.
